# Development of a high-throughput SARS-CoV-2 antibody testing pathway using dried blood spot specimens

**DOI:** 10.1177/0004563220981106

**Published:** 2020-12-26

**Authors:** Stuart J Moat, Wioleta M Zelek, Emily Carne, Mark J Ponsford, Kathryn Bramhall, Sara Jones, Tariq El-Shanawany, Matt P Wise, Annette Thomas, Chloe George, Christopher Fegan, Rachael Steven, Russell Webb, Ian Weeks, B Paul Morgan, Stephen Jolles

**Affiliations:** 1 Wales Newborn Screening Laboratory, Department of Medical Biochemistry, Immunology and Toxicology, University Hospital of Wales, Cardiff, Wales, UK; 2 School of Medicine, Cardiff University, Cardiff, Wales, UK; 3 Systems Immunity University Research Institute and Dementia Research Institute, Cardiff University, Cardiff, UK; 4 Immunodeficiency Centre for Wales, University Hospital of Wales, Cardiff, UK; 5 Division of Infection, Inflammation and Immunity, School of Medicine, Cardiff University, Cardiff, UK; 6 Weqas, Cardiff and Vale University Health Board, Cardiff, UK; 7 Adult Critical Care, University Hospital of Wales, Cardiff, UK; 8 Welsh Blood Service, Ely Valley Road, Talbot Green, Pontyclun, UK; 9 Department of Haematology, University Hospital of Wales, Cardiff, Wales, UK; 10 College of Biomedical and Life Sciences, Cardiff University, Cardiff, UK

**Keywords:** Dried blood spots, SARS-CoV-2, antibodies, enzyme-linked immunosorbent assay, COVID-19

## Abstract

**Background:**

Serological assays for Severe Acute Respiratory Syndrome Coronavirus 2 (SARS-CoV-2) have roles in seroepidemiology, convalescent plasma-testing, antibody durability and vaccine studies. Currently, SARS-CoV-2 serology is performed using serum/plasma collected by venepuncture. Dried blood spot (DBS) testing offers significant advantages as it is minimally invasive, avoids venepuncture with specimens being mailed to the laboratory.

**Methods:**

A pathway utilizing a newborn screening laboratory infrastructure was developed using an enzyme-linked immunosorbent assay to detect IgG antibodies against the receptor-binding domain of the SARS-CoV-2 spike protein in DBS specimens. Paired plasma and DBS specimens from SARS-CoV-2 antibody-positive and -negative subjects and polymerase chain reaction positive subjects were tested. DBS specimen stability, effect of blood volume and punch location were also evaluated.

**Results:**

DBS specimens from antibody-negative (*n* = 85) and -positive (*n* = 35) subjects and polymerase chain reaction positive subjects (*n* = 11) had a mean (SD; range) optical density (OD) of 0.14 (0.046; 0.03–0.27), 0.98 (0.41; 0.31–1.64) and 1.12 (0.37; 0.49–1.54), respectively. An action value OD >0.28 correctly assigned all cases. The weighted Deming regression for comparison of the DBS and the plasma assay yielded: *y* = 0.004041 + 1.005*x*, *r* = 0.991, Sy/*x* 0.171, *n* = 82. Extraction efficiency of antibodies from DBS specimens was >99%. DBS specimens were stable for at least 28 days at ambient room temperature and humidity.

**Conclusions:**

SARS-CoV-2 IgG receptor-binding domain antibodies can be reliably detected in DBS specimens. DBS serological testing offers lower costs than either point of care or serum/plasma assays that require patient travel, phlebotomy and hospital/clinic resources; the development of a DBS assay may be particularly important for resource poor settings.

## Introduction

Serological testing for Severe Acute Respiratory Syndrome Coronavirus 2 (SARS-CoV-2) is important clinically, in the selection of convalescent plasma donors, understanding durability of antibody responses, to assess the impact of a second or subsequent surge, to monitor antibody production when vaccines become available and in seroprevalence studies. A number of point of care tests (PoCT) and larger scale commercially available immunoassay platforms have been developed for serological assessment of SARS-CoV-2 antibodies in plasma/serum.^1^,^2^ In April 2020, due to urgency and demand, the FDA issued an umbrella Emergency Use Authorization (EUA) for COVID-19 serological testing, allowing these assays to be released without review by the FDA.^2^ However, this umbrella EUA was revoked in July 2020 and individual EUAs now have to be issued that meet the requisite EUA statutory criteria.^3^ The choice of assay and its performance characteristics are therefore critical to address the potential categories of use. However, the utility of these plasma/serum tests has not been fully defined, with few studies having been published using large patient cohorts.^4^

There is an urgent need to develop sensitive and specific cost-effective assays which have a high sample volume throughput. Such assays would allow collection of more granular information to inform modelling regionally and policy on lock down measures, allow testing ahead of planned hospital procedures and facilitate seroprevalence studies in care home residents, health and social care workers and other priority groups. The scalability of conventional approaches is limited by the parallel requirement for a huge increase in phlebotomy workforce (estimated 5–10 blood venepunctures per hour per phlebotomist – at the lower end of the range if needing to use personal protective equipment).

Dried blood spot (DBS) specimens have been used both to screen babies for phenylketonuria and to monitor their dietary treatment since the 1960s. The use of DBS specimens to monitor patients is widely favoured due to the simplicity and convenience of collecting blood from a finger prick onto a filter paper collection device in the patient’s home and mailing the specimen directly to the laboratory for analysis.^5^ DBS collection methods have also proven useful for serology testing of hepatitis and human immunodeficiency virus (HIV) in resource-limited settings.^6–8^ Furthermore, in a recent cost evaluation study of the use of DBS specimen collection compared to conventional sampling, it was shown that DBS sampling was associated with significant cost reductions.^9^

We propose DBS testing for anti-SARS-Cov-2 antibodies using a ‘kit’ containing instructions on using a lancet to obtain capillary blood, a blood spot filter paper collection device and a return mail envelope, this would enable testing without an appointment or the need to travel to a health-care facility. The costs of DBS serological testing are lower than either lateral flow PoCT assays or laboratory assays that require patient travel, phlebotomy and hospital/clinic resources, making the development of a DBS assay for SARS-CoV-2 antibodies advantageous in resource poor settings.

Accordingly, a scaleable and cost-effective assay protocol was developed to elute and detect SARS-CoV-2 receptor-binding domain (RBD)-specific IgG antibodies in DBS specimens using an enzyme-linked immunosorbent assay (ELISA)-based assay. The aim of this study was to characterize the performance of this assay protocol in DBS specimens using an existing national newborn screening laboratory infrastructure.

## Materials and methods

### Reagents and consumables

All chemicals, except where otherwise stated, were obtained from either Fisher Scientific UK (Loughborough, UK) or Sigma Aldrich (Gillingham, UK) and were of analytical grade. PerkinElmer 226 filter paper (PerkinElmer, Turku, Finland) was used for all sample collections and experimental preparations. DBS subpunches were obtained using a DBS puncher (PerkinElmer, Turku, Finland) using 6 and 3.2 mm diameter punch heads.

### Test specimens

Specimens used in the assay evaluation and analysis were either anonymized residual samples which would otherwise have been discarded or obtained from participants who provided consent for additional DBS testing with Health Research Authority ethical approval – IRAS Project ID 284129. Patient specimens were classified on the basis of a positive or negative result as assessed by one of the following SARS-CoV-2 antibody detection methods: Abbott Diagnostics IgG, PoCT (IgG/IgM-Healgen Scientific # GCCOV-402a) or Euroimmun IgG assays. An additional 11 DBS specimens were obtained from polymerase chain reaction (PCR) positive cases. The duration from symptom onset and specimen collection was not recorded.

### Direct ELISA to detect RBD immunoglobulin G antibodies

An in-house direct ELISA was performed as previously described^10^,^11^ with the following modifications. Maxisorp (Nunc, Loughborough, UK) 96-well plates were coated with RBD protein (recombinantly generated in a mammalian expression system, in-house) at 0.5 µg/mL in bicarbonate buffer, pH 9.6 at 4°C over-night; wells were blocked (1 h at room temperature [RT] with 3% w/v non-fat dried milk powder in phosphate-buffered saline containing 0.1% Tween-20 [PBS-T]) and washed in PBS-T. Dilutions of patient serum/plasma (1 in 50 in PBS-T containing 1% non-fat milk) were added to coated wells and incubated for 2 h at RT. Wells were washed three times with PBS-T then incubated (1 h, RT) with a secondary antibody (donkey anti-human IgG F(ab′)_2_-horseradish peroxidase; Jackson ImmunoResearch, # 709-036-149, Ely, UK) for 1 h at RT. After washing (3×), plates were developed using *O*-phenylenediamine dihydrochloride and the optical density (OD) measured at 492 nm. This assay was initially validated for the measurement of serum/plasma samples and then further validated for DBS eluates. The DBS manual ELISA was then transferred onto an automated platform (DSX®, Dynex Technologies).

### Assay optimization

To optimize the conditions for the ELISA analysis of DBS specimens, we compared different elution buffers and DBS subpunch size. Residual whole blood EDTA specimens from SARS-CoV-2 negative and positive subjects were applied (100 *µ*L aliquots) to filter paper. Specimens were dried over-night at ambient temperature. Subpunches of 6 and 3.2 mm diameters were taken from the DBS specimens, placed into 2 mL 96-well plates (Waters # 186002482). Buffers comprising PBS-T containing non-fat milk (1–5%) or bovine serum albumin (0.5%) were added in varying volumes (200–500 µL). The plates were then covered and shaken at RT for up to 3 h. In addition, we assessed the effect of adding sodium azide and hydrogen peroxide to the eluting buffers to inhibit the background red cell peroxidases. Preliminary investigations demonstrated that incubating a 6-mm subpunch in 300 *µ*L of PBS containing 0.1% Tween-20 and 5% non-fat milk protein for 1 h gave the optimum OD signal in eluates from positive samples and the lowest background OD signal in the negative samples. The protocol was aligned with the existing national newborn screening laboratory equipment and work flows using semi-automated subpunching equipment, allowing two 96-well plates to be prepared per hour (DELFIA DBS Puncher, PerkinElmer), with same day elution (1 h) and transfer to 96-well ELISA plates.

**Figure 1.**
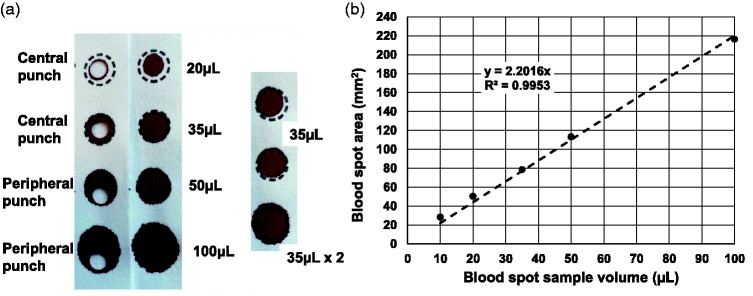
(a) Effect of sample volume on blood spot diameter. A whole blood EDTA sample was applied to filter paper collection devices at the volumes shown. Subpunches (6 mm) were taken from the central and peripheral locations. (b) Relationship between the volume of blood applied to filter paper collection device and the DBS area. Results are the mean of five replicates.

To determine the optimal volume of buffer used to elute the antibodies from the DBS specimens in order to give comparable OD results to the paired plasma/serum specimens (diluted 1 in 50), we calculated the volume of serum/plasma in a 6-mm punch. Whole blood EDTA samples (*n* = 8) were pooled and sample volumes of 10, 20, 35, 50 and 100 *µ*L applied to filter paper (*n* = 5) using a calibrated pipette. The diameters of the various DBS specimens were measured (using a magnifying ruler) and the area of the DBS was calculated (*πr*^2^) and plotted against the blood volume applied to the filter paper collection device (Figure 1).

Recovery of SARS-CoV-2 IgG antibodies from DBS specimens was assessed using a series of whole blood EDTA specimens (*n* = 7); specimens were gently mixed, 100 *µ*L aliquots dispensed onto filter paper and dried over-night at ambient temperature. Whole blood lysates were prepared from the same samples by subjecting a 200 *µ*L aliquot of whole blood to an over-night freeze (–20°C) to lyse the red cells. The remaining portion of the specimen was then centrifuged, the plasma removed and stored at 4°C prior to analysis. DBS specimens were punched in duplicate and analysed in a single analytical batch with paired plasma and whole blood lysate specimens.

Assay precision was assessed using antibody-negative quality control (QC) and antibody-positive QC specimens, generated by pooling residual anonymized EDTA whole blood specimens from antibody-positive and -negative subjects. Aliquots (100 *µ*L) of whole blood were dispensed onto filter paper and dried over-night at ambient temperature. DBS QC material was then stored desiccated at –20°C.

The utility of five commercially available SARS-CoV-2 antibody assays for DBS eluate testing were also explored: Siemens total antibody (IgG/A/M), Siemens IgG (Siemens, Frimley, UK), Ortho Diagnostics IgG (Ortho Clinical Diagnostics, Bridgend, UK), Abbott Diagnostics IgG (Abbott, Maidenhead, UK) and Roche IgG (Roche Diagnostics, Burgess Hill, UK). The capacity of each assay to detect SARS-CoV-2 antibodies in DBS specimens using our elution protocol was assessed.

Variability in DBS specimen collection and subpunch location can affect analyte results. To explore this, the effect of blood sample volume and non-homogeneity in the DBS specimens on the measurement of SARS-CoV-2 IgG antibodies was assessed by preparing pooled residual EDTA blood samples from antibody-positive subjects (*n* = 8). Aliquots of 20, 35, 50 and 100 *µ*L of the pooled blood were dispensed onto filter paper and dried over-night at ambient temperature. To examine the effect of double layering of blood onto the filter paper collection device, 35 *µ*L of whole blood was applied to the filter paper and then a further 35 *µ*L was re-applied immediately. Five subpunches from the different DBS size categories were analysed by punching once from the centre and twice from the edge. Only central punches were taken from the 10 and 35 *µ*L specimens (area too small for >1 punch).

### Analysis of paired plasma and DBS specimens

EDTA samples were collected as part of routine Hospital staff SARS-CoV-2 antibody testing. EDTA samples were gently mixed and 100 *µ*L of blood was applied onto the filter paper using a pipette. The remainder of the EDTA blood specimen was centrifuged and the plasma removed for analysis using the in-house ELISA. Contemporaneous serum/plasma samples were also collected and analysed using the lateral flow PoCT device (IgG/IgM-Healgen Scientific, Houston, USA).

### Assessment of SARS-CoV-2 antibody stability in DBS specimens

Residual whole blood EDTA specimens from SARS-CoV-2 antibody-positive subjects (*n* = 12) were pooled. Replicate DBS specimens were prepared by applying 100 *µ*L volumes to filter paper, dried over-night at ambient temperature and humidity. Specimens were then stored either at ambient humidity, within a sealed foil bag containing a desiccant (low humidity) or in a sealed box containing wet paper towels (high humidity), at the following temperatures: –20°C, +4°C, RT (∼25°C) and 40 °C for between 1 and 28 days. At various time intervals (Table 1), samples were removed and stored desiccated in sealed foil bags at –20°C prior to analysis in duplicate at the end of the study. Results were calculated as the % residual OD, using DBS specimens that were stored desiccated at –20°C, during the study period as the control (i.e. 100%) and analysed alongside those specimens subjected to the various heat and humidity conditions.

**Table 1.** Stability of COVID-19 antibodies under various conditions of temperature and humidity.

	Length of storage (days)						
Storage conditions	0	1	3	7	14	21	28
–20°C, low humidity	100	93	100	103	101	97	97
–20°C, ambient humidity	100	107	109	99	109	97	90
+4°C, low humidity	100	99	102	104	105	92	102
+4°C, ambient humidity	100	105	98	104	97	92	92
RT, low humidity	100	107	103	103	99	96	102
RT, ambient humidity	100	99	104	103	102	97	93
RT, high humidity	100	97	102	102	91	92	94
40°C, low humidity	100	108	101	96	95	90	91
40°C, ambient humidity	100	107	97	96	94	90	97
40°C, high humidity	100	81	65	57	45	29	27

DBS specimens were stored at the various temperatures and conditions. At the various time intervals, samples were removed and stored desiccated in sealed foil bags at –20°C prior to analysis in duplicate at the end of the study. Results shown are % residual OD, using DBS specimens that were stored desiccated at –20°C, during the study period as the control (i.e. 100%) and analysed alongside those specimens subjected to the various heat and humidity conditions. The mean (SD) OD for the 100% control was 1.52 (0.14).

### Statistical analysis

Analysis of differences between groups was carried out using a Kruskal–Wallis test with a Tukey’s Multiple Comparison Test. The action value OD for positivity was calculated for DBS samples as the mean + 3 SD of the results obtained from antibody-negative subjects. All analyses were performed using SPSS v21.

## Results

### Relationship of DBS area to blood volume

The relationship between DBS area and volume was linear (*r* = 0.998) and the gradient of the slope was *y* = 2.2016*x* (Figure 1). Thus a 6-mm subpunch (area 28.26 mm^2^) corresponds to a whole blood volume of 12.84 *µ*L and approximately 5.8–6.4 *µ*L of plasma/serum (assuming an average haematocrit of 45–50% in adult subjects). Using an elution buffer volume of 300 *µ*L for a single 6-mm subpunch, it is estimated that the serum component is diluted by a factor of 1 in 47 to 1 in 51, which is comparable with the optimal dilution for plasma/serum samples in the assay.

### Assay performance

For antibody recovery studies, the mean (SD and range) OD responses for paired DBS, plasma and whole blood lysates (*n* = 7) was 1.39 (0.27; 0.97–1.64), 1.29 (0.34; 0.77–1.65) and 1.27 (0.33; 0.74–1.6), respectively. The mean (SD; range) extraction efficiency (%) of plasma antibodies from DBS specimens using the optimized buffer was 109 (10.6; 99–125). The intra-assay (*n* = 10) and interassay (*n* = 8) coefficient of variations for the antibodies in the QC specimens, respectively, were as follows: negative (0.22 OD), 8.7% and 12.6%, and positive (1.58 OD) 3.3% and 10.6%.

### Analysis of DBS from SARS-CoV-2 antibody-positive and -negative subjects

**Figure 2.**
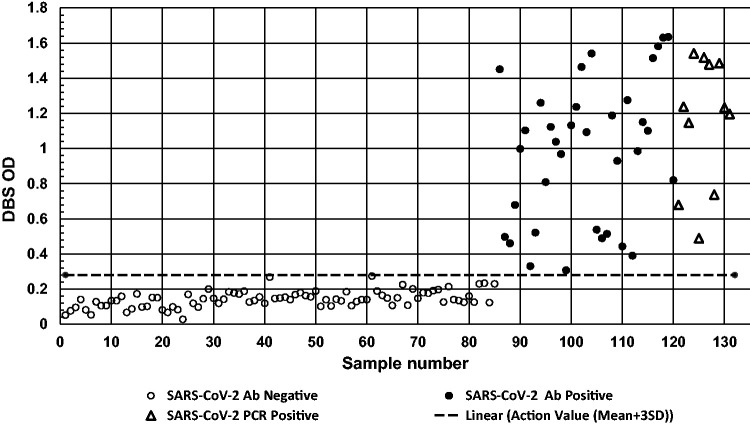
Performance of the DBS SARS-CoV-2 IgG ELISA for specimens from SARS-CoV-2 antibody negative subjects (*n* = 85), SARS-CoV-2 antibody-positive subjects (*n* = 35) and SARS-CoV-2 PCR positive subjects (*n* = 11). The dotted line represents the action value for positivity on the DBS ELISA (OD > 0.28).

DBS specimens from SARS-CoV-2 antibody-negative subjects (*n* = 85) had a mean (SD; range) OD of 0.14 (0.046; 0.03–0.27), while SARS-CoV-2 antibody-positive cases (*n* = 35) had a mean (range) OD of 0.98 (0.41; 0.31–1.64). Eleven subjects who tested positive for COVID-19 by PCR had a mean OD response of 1.1 (0.37; 0.49–1.54) (Figure 2). The action value OD for the SARS-CoV-2 antibody-positive DBS samples was calculated as the mean + 3 SD of the results obtained from antibody-negative subjects (OD > 0.28) and correctly classified all cases (Figure 2).

**Figure 3.**
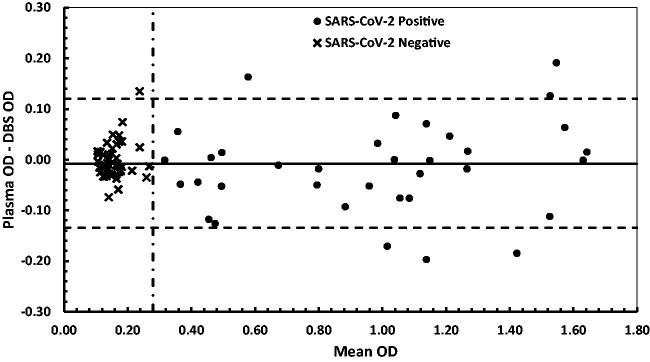
Bland–Altman plot showing the absolute difference for SARS-CoV-2 IgG antibody OD’s between the plasma specimens analysed by the in-house ELISA and in paired DBS specimen eluates analysed by the in-house ELISA. The solid horizontal line denotes the mean bias between the two methods (–0.008 OD). The dashed horizontal lines indicate the 95% limits of agreement. The dashed vertical line denotes the action value for positivity on the DBS ELISA (>0.28). The weighted Deming plot yielded a slope of 1.005 (95% CI: 0.9573–1.053), intercept of 0.004 (95% CI: –0.007 to 0.015), an Sy/*x* of 0.171 and a correlation coefficient (*r*) of 0.991. Paired plasma and DBS specimens used were from SARS-CoV-2 negative subjects (*n* = 47) and positive antibody subjects (*n* = 35).

**Figure 4.**
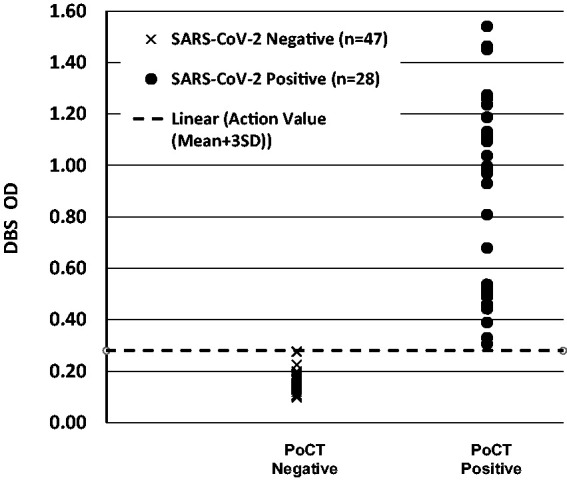
Concordance between the DBS ELISA and the Healgen Scientific PoCT (IgG/IgM) lateral flow device using paired DBS and serum specimens from SARS-CoV-2 negative subjects (*n* = 47) and positive antibody subjects (*n* = 28). The dotted line represents the action value for positivity on the DBS ELISA (OD > 0.28).

A Bland–Altman plot of the absolute difference between SARS-CoV-2 IgG antibodies in plasma specimens analysed by the in-house ELISA and in paired DBS specimen eluates analysed by the DBS ELISA, demonstrated a mean bias between the two methods of –0.008 OD (Figure 3). Deming regression analysis (Analyze-It, Leeds, UK) revealed an agreement between the assays for SARS-CoV-2 IgG antibodies in the paired plasma and DBS specimens, which demonstrated a slope (95% CI) of the regression line of 1.005 (0.9573–1.053), intercept (95% CI) of 0.004 (–0.007 to 0.015), an Sy/*x* of 0.171 and a correlation coefficient (*r*) of 0.991. Serum samples analysed using the Healgen Scientific PoCT lateral flow device (detects antibodies to both the viral spike and nucleocapsid proteins) and the paired DBS eluates analysed using the in-house ELISA gave concordant results (Figure 4).

### Comparison of DBS eluates analysed by the in-house ELISA versus commercial serological assays

**Figure 5.**
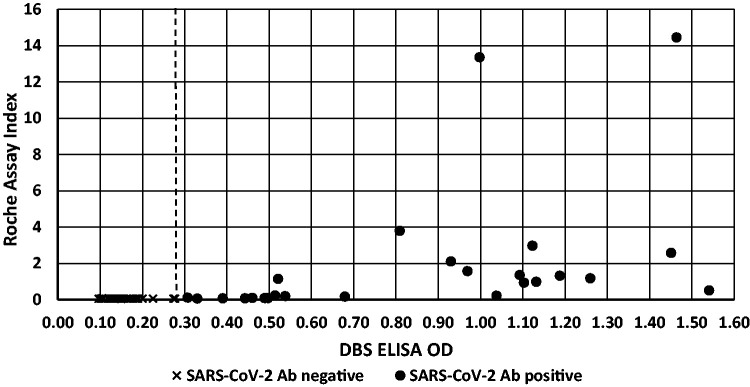
Concordance between the DBS ELISA and Roche commercial IgG serological assay using DBS specimen eluates from SARS-CoV-2 negative subjects (*n* = 44) and positive antibody subjects (*n* = 25). The dotted line represents the action value for positivity on the DBS ELISA (OD > 0.28).

High background signals were obtained using DBS eluates on the Ortho Diagnostics IgG assay and both the Siemens total antibody and IgG assays; as a consequence, these assays were unable to distinguish between SARS-CoV-2 antibody-positive and -negative samples (data not shown). The Roche assay gave adequate separation in results between the positive and negative QC samples. However, in a subset of DBS specimens from SARS-CoV-2 antibody-negative (*n* = 44) and -positive cases (*n* = 25), the Roche assay was not sensitive enough to detect antibodies in the majority of the DBS eluates from antibody-positive cases (Figure 5).

### Effect of DBS size and punch location

OD values were significantly affected by DBS size and punch location (*P* = 0.002). Smaller blood spot volumes (20 and 35 *µ*L) produced significantly lower OD values (10% and 16%, respectively) when compared to the 100 *µ*L DBS with a peripheral punch (*P* < 0.05). Peripheral punches from layered specimens (double spotted 35 *µ*L DBS) produced significantly higher OD values than those obtained from 20, 35 and 50 *µ*L central punches (11%, 17% and 11%, respectively) (*P* < 0.05). The OD values obtained using a peripheral punch from the 100 *µ*L and the double spotted 35 *µ*L DBS specimens were 11% and 13.0% higher (respectively) than those obtained from a 50 *µ*L central punch (*P* < 0.05). A positive bias was observed for the antibody ODs obtained using a peripheral punch versus a central punch, however, this did not reach statistical significance.

### DBS specimen stability studies

Both humidity and temperature affected the stability of the antibodies in DBS specimens (Table 1). At RT with low or ambient humidity, antibodies were stable for at least 28 days. The same was observed at +4°C and –20°C with both ambient and low humidity conditions. Specimens stored at +40°C with low or ambient humidity were stable for up to 7 days. However, antibodies degraded rapidly when stored at +40°C and at high humidity, with decreases of 35%, 55% and 73% being observed at days 3, 14 and 28, respectively.

## Discussion

In this work, we describe the systematic optimization of the elution of SARS-CoV-2 RBD IgG antibodies from DBS specimens for analysis using an optimized in-house high-throughput ELISA. Our protocol builds upon a proven infrastructure of newborn blood spot screening testing and allows the cost-effective use of equipment and expertise that currently exist in these laboratories.

Several studies have recently examined the utility of DBS SARS-CoV-2 antibody testing.^12–17^ However, many of these studies suffer from small sample sizes^12,14,15^ or use sample punching/sample extraction procedures that do not make the assay amenable for rapid high-throughput testing.^12,15,16^ Furthermore, none of these studies have linked testing with the established newborn DBS screening laboratory infrastructure.

The development of high-throughput DBS anti-SARS-CoV-2 antibody testing which links with the newborn DBS screening laboratories is of particular value for three main reasons. First, health-care workers such as phlebotomists, nurses or doctors are not required to take blood, reducing the risk of possible COVID-19 transmission to or from health-care workers, and the sample can easily be taken in the home; this is particularly important for those vulnerable groups who may be shielding. Second, the method does not require blood collection tubes or syringes, and given our specimen stability findings, the DBS can be sent via the mail/postal system, enabling testing at distance, where blood collection resources are limited, and at scale. DBS specimens pose a minimal biohazard risk due to the fact that viruses on the cotton filter paper matrix loose infectivity upon drying and so are not readily transmissible.^18–20^ The send-in approach automatically links the testing to centralized laboratory platforms, which feed results directly into existing electronic pathology systems, which negates the need for further software development and additional IT infrastructure resource required when using PoCT lateral flow devices at home. Third, the cost of collecting the specimen is low, which becomes increasingly important in scenarios where many specimens are needed (e.g. when testing specific population cohorts such as students, school teachers, nursing home residents or groups of health-care workers in seroprevalence studies) as the cost of obtaining the specimen is significantly greater than the cost of the test itself.

The DBS OD values obtained in our assay for SARS-CoV-2 antibody-negative and -positive subjects were comparable to the plasma OD values observed in our assay and in previous studies on which our ELISA is based.^10,11^ The action value for positivity (0.28) correctly classified all cases. The OD values observed in the antibody and PCR positive cases demonstrated complete separation from the antibody-negative subjects. However, further work is required to validate the OD cut-off for positivity, using paired DBS and plasma/serum specimens from a larger number of mild and severe COVID-19 patients, with confirmed positive PCR test results and to assess the impact of duration from symptom onset on antibody concentrations.

In addition, we demonstrated good agreement between the OD values obtained for paired DBS and plasma specimens, confirming excellent recovery of the antibodies from DBS specimens. This finding confirms that of Morley et al., using a similar ELISA format.^16^ To further characterize the performance of our DBS assay, we compared the OD values obtained for DBS specimens analysed using the ELISA to the results from paired serum results obtained using the Healgen Scientific PoCT (IgG and IgM) lateral flow device (detects antibodies to both the spike and nucleocapsid proteins). The results demonstrated good concordance between paired specimens.

Our assay detects the RBD of the virus spike protein, whereas many of the commercial assays detect the nucleocapsid protein. Current assays are evolving with an emphasis on those assessing antibodies to the RBD protein as these appear to correlate better with neutralizing antibodies and may be more sensitive in mild disease settings.^21^ The open nature of ELISA allows for optimization to different sample types (e.g. saliva), which is more challenging when using the ‘black box’ commercial assays. It also permits further flexibility in evolution of testing e.g. SARS-CoV-2 IgA from serum/plasma or saliva^22^ or the detection of antibodies to the nucleocapsid, RBD and spike proteins in a single assay.^17^

Furthermore, assay performance will be improved when certified reference material for anti-SARS-CoV-2 antibody calibrators become available and enable standardization of quantitative results. Indeed, efforts are currently being made to develop calibrator material for assay standardization.

The volume of blood applied to the filter paper collection device, and the location of the subpunch (peripheral versus central) can have a significant impact on the result obtained for certain metabolites.^23^ We examined the impact of DBS specimen volume and punch location on antibody OD responses and demonstrated that these factors have a small but significant effect on the results obtained. These findings are consistent with previous reports for biomarkers quantified in DBS specimens and highlight the importance of obtaining good quality specimens for analysis to ensure accurate test results.^23–25^

DBS specimens need to be transported to the laboratory (usually via the mail service) for analysis and during this process (2–5 days) specimens can be exposed to extreme environmental conditions. Assay protocols for plasma/serum SARS-CoV-2 antibodies recommend that samples should be heat treated (60 min at 56°C) to inactivate the virus.^10,11^ It has been shown that heat treated samples gave comparable OD values to that of non-heat treated samples^10^ (and in-house data; not shown) demonstrating that COVID-19 antibodies are relatively stable. However, no previous studies have assessed the long-term stability of SARS-CoV-2 IgG antibodies in DBS specimens at various temperatures and humidity. We have shown that antibodies in DBS specimens are stable at ambient and lower temperatures. However, antibodies degraded rapidly when stored at +40°C with high humidity. Our findings are comparable to those found in studies assessing the stability of HIV antibodies in DBS specimens at various temperatures and humidty.^26^ While these data give confidence that standard mailing in temperate climates will have little impact on DBS measurements, we recommend that DBS specimens should be protected from moisture as much as possible and should be stored desiccated for the short term at 4°C, prior to analysis in the laboratory. On the basis of our findings, it is likely that long-term stability can be achieved using desiccants and storage temperatures of –20°C or lower. An incidental finding during the stability study was the observation that those subpunches taken from specimens subjected to both high humidity and temperature, remain dark red, following the elution step, whereas those taken from appropriately stored specimens turn from red to white following the elution step. Therefore, the quality of the specimen received for analysis (and the validity of the result) can be assessed visually following the elution step and those samples where the blood has not fully eluted from the subpunch, should be rejected.

In conclusion, this study demonstrates the utility of DBS specimen collection for SARS-CoV-2 antibody detection using an ELISA as a cost-effective and efficient approach for large-scale antibody testing and may be particularly important for resource poor settings.
